# Reliability-Based Design Optimization for Polymer Electrolyte Membrane Fuel Cells: Tackling Dimensional Uncertainties in Manufacturing and Their Effects on Costs of Cathode Gas Diffusion Layer and Bipolar Plates

**DOI:** 10.3390/molecules29184381

**Published:** 2024-09-14

**Authors:** Neil Vaz, Kisung Lim, Jaeyoo Choi, Hyunchul Ju

**Affiliations:** Department of Mechanical Engineering & BK21 FOUR Education and Research Team for Overcoming Mechanical Challenges in Carbon Neutrality, Inha University, 100 Inha-ro Michuhol-Gu, Incheon 22212, Republic of Korea

**Keywords:** polymer electrolyte fuel cell, reliability-based design optimization, dimensional uncertainties, multi-layer perceptron, particle-swarm optimization, dimensional tolerance, Monte Carlo simulation, dynamic Kriging surrogate, deterministic design optimization

## Abstract

Polymer Electrolyte Membrane Fuel Cells (PEMFCs) have emerged as a pivotal technology in the automotive industry, significantly contributing to the reduction of greenhouse gas emissions. However, the high material costs of the gas diffusion layer (GDL) and bipolar plate (BP) create a barrier for large scale commercial application. This study aims to address this challenge by optimizing the material and design of the cathode, GDL and BP. While deterministic design optimization (DDO) methods have been extensively studied, they often fall short when manufacturing uncertainties are introduced. This issue is addressed by introducing reliability-based design optimization (RBDO) to optimize four key PEMFC design variables, i.e., gas diffusion layer thickness, channel depth, channel width and land width. The objective is to maximize cell voltage considering the material cost of the cathode gas diffusion layer and cathode bipolar plate as reliability constraints. The results of the DDO show an increment in cell voltage of 31 mV, with a reliability of around 50% in material cost for both the cathode GDL and cathode BP. In contrast, the RBDO method provides a reliability of 95% for both components. Additionally, under a high level of uncertainty, the RBDO approach reduces the material cost of the cathode GDL by up to 12.25 $/stack, while the material cost for the cathode BP increases by up to 11.18 $/stack Under lower levels of manufacturing uncertainties, the RBDO method predicts a reduction in the material cost of the cathode GDL by up to 4.09 $/stack, with an increase in the material cost for the cathode BP by up to 6.71 $/stack, while maintaining a reliability of 95% for both components. These results demonstrate the effectiveness of the RBDO approach in achieving a reliable design under varying levels of manufacturing uncertainties.

## 1. Introduction

Fuel cells (FCs) are considered to be a key enabling technology for the emerging hydrogen economy [1]. These devices electrochemically convert fuels such as hydrogen and oxygen to generate electricity. FCs are silent in operation with zero emission of harmful pollutants and can generate electricity if the source of fuel is supplied. FCs are generally classified based on the conducting electrolyte used, operating temperature and the feasible performance region [2]. PEMFCs are considered to be one of the most promising sources of energy conversion devices that would perhaps replace the internal combustion engines [3]. PEMFCs are particularly well suited for stationary and mobile applications. In PEMFCs, the electrochemical reaction of hydrogen along with oxygen to form water is divided into the partial reactions of oxidation and reduction by incorporating a proton-conducting membrane between the anode and cathode electrodes. PEMFCs are typically operated at temperatures ranging between 50 °C and 80 °C and have high power density and low degradation rates [4]. 

PEMFCs have received immense attention due to their wide range of application. Their application in the real world ranges from industrial scale systems for power backup to mobile power for trains, buses, heavy duty trucks and material handling systems [5]. However, in recent years, the widespread implementation of PEMFCs has been restricted due to a rise in manufacturing costs and issues pertaining to durability in fuel cell designs [6,7]. Therefore, it is imperative to work over the design aspects of fuel cells. The modern fuel cell market is highly competitive and requires engineers to come up with designs that are inexpensive and highly reliable. The design process is quite intricate and is largely focused on producing products that are characterized by being inexpensive, of excellent quality and of great durability. The manufacturer’s data sheets fail to specifically address all of the various actual aspects that contribute to the complexity and high nonlinearity of PEMFC models. As such, accurate optimization methods are necessary to identify and determine these unidentified elements in the fuel cell model [8]. The modern design process is based on complex simulation models that can support complexity and fidelity to accomplish the aforementioned objectives and are often referred to as a simulation-based design approach [9].

Over the past few decades, the computational speed of computers has increased exponentially leading to the development and application of large-scale simulation models. Simulation tools like computational fluid dynamics (CFD) and finite element analysis (FEA) have seen large growth and are able to represent an actual physical system. This has provided design engineers with a wide range of opportunity to come up with improved and optimal design strategies. To create high-quality design models, the engineering community of today has been using optimization to a greater extent. These design models have been demonstrated to be cost effective and have acceptable performance abilities. In most cases, engineers consider the design variables to be deterministic during engineering design optimization, and the process of attaining an optimal design on this basis is referred to as DDO. 

PEMFCs have been optimized using numerical analysis to improve $V_{cell}$ by considering the design variables to be deterministic. Song et al. [10] optimized the cathode catalyst layer by considering the one-dimensional macro homogenous model where the four design parameters included Nafion content, void volume fraction, thickness and the amount of platinum (Pt) loading. Grigoriev et al. [11] optimized the geometry of the BP and GDL of a high-temperature fuel cell and provided insight into the effects of these parameters on $V_{cell}$. Kim et al. [12] conducted a comprehensive study considering metallic BPs and evaluated the effects of channel-to-rib width ratio, draft angle, inner fillet radius and clamping pressure. The study reveals that the GDL intrusion is highly influenced by the channel-to-rib width ratio and draft angle, which in turn affects the pressure drop within the channels. To address the effects of durability in fuel cell hybrid electric vehicles (FCHEV), Tang et al. [13] proposed the degradation adaptive energy management strategy (EMS) to address the effects of durability in fuel cell hybrid electric vehicles(FCHEV) that is based on the deep deterministic policy gradient model. The proposed degradation adaptive EMS optimizes energy consumption and durability by adapting to the current state of health of the FCHEV powertrain. It outperforms existing EMS models and reduces fuel cell stack start–stop cycles, enhancing component longevity. However, a typical engineering process consists of various uncertainties. Products manufactured based on a DDO approach will have varying performance characteristics and a high risk of failure as they do not consider uncertainties in them. In real world scenarios, uncertainties may often arise due to external operating conditions, variations in parameters such as dimensions or material properties, model uncertainties and errors associated with the simulation tools used for simulation-based designs and many more. When the uncertainties are taken into consideration, some types of constraints such as the initial condition and $V_{cell}$ may be violated. Therefore, to avoid the risk for any given product to fail, these uncertainties must be considered during the optimization process. 

Optimization methods that consider the uncertainties in design variables and solve an optimization problem with reliability constraints are referred to as RBDO [14,15]. With RBDO, the designers are able to determine optimal designs that would meet target reliability measures that would achieve satisfactory levels of performance measures and constraints. RBDO has been widely used in the field of structural designs and fluid–structure interaction problems, magnetic energy storage systems and multi-body dynamic systems. However, to date there has been no research presented where RBDO has been used to optimize the design variables for a PEMFC. 

PEMFCs are highly complex systems that consist of several components. These components have varying material properties and manufacturing tolerances. Any minor changes in the component properties and dimensions along with PEMFC operating conditions such as temperature, humidity and pressure will affect the PEMFC performance. Hence, variability must be considered during the design stage itself. RBDO engineers consider the input design variables of a probability distribution and carry out optimization to determine an optimal design solution [16]. The designs thus obtained are reliable and have a very low chance of failure. Dimensional tolerances, which refer to the allowable deviation from the specified dimension, are a critical part of the manufacturing of PEMFC components [17,18,19]. Intricate components are expensive to manufacture and must meet strict dimensional tolerance levels. Small deviations in dimensions can cause a significant reduction in performance and costs. For example, the membrane electrode assembly (MEA) thickness has to be precisely controlled so that the flow of reactant gases reaches the catalyst layer (CL). Likewise, $\delta_{gdl}$ and $d_{ch}$ must be controlled to make sure that the reactant gases and flow of liquid water is managed well within the cell. 

The dimensional tolerance considered during manufacturing plays a pivotal role in increasing the costs of PEMFCs [20]. The process of manufacturing PEMFC components is highly complex and requires high precision. In addition, the use of expensive and high-quality materials to meet high-dimensional tolerance levels leads to an increase in the overall cost of PEMFCs. A fuel cell stack consists of hundreds of single cells that are arranged in series and generate the required power and voltage for operation. Highly precise manufacturing accuracy in BPs and GDLs is required to obtain uniform contact pressure and electrochemical reactions in the stacks [21,22,23]. However, due to the manufacturing process, errors arise in shape, dimensions and assembly that are inevitable. The stamping process is the most preferred choice for manufacturing BPs [24,25]. During the stamping process, highly localized stamping forces are induced while channels are formed, leading to errors in the dimensions of channel height and width [26,27]. In addition, dimensional variation in GDL and BPs would also lead to assembly errors and causes failure of the systems [28]. Thus, there is a need to consider these errors during the optimization stage.

The GDLs serve as a medium for distributing the reactant gases and are generally made of carbon fibers. On the other hand, BPs are employed within the fuel cell stack to conduct electricity and separate individual cells. The amount spent on materials is significantly impacted by the dimensional tolerance needed throughout the production process. It will take high-quality materials with reliable properties to achieve tighter tolerance. Also, the material wastage for manufacturing GDLs and BPs with tighter tolerance levels will be high. This is due to the fact that parts must be scrapped or reworked when they do not meet the required dimensional tolerance. Likewise, for looser tolerance there is a possibility of using low-cost material with wide variations in thickness. This may result in a lower material cost with additional processing steps required and a compromise in $V_{cell}$. Overall, the effect of dimensional tolerance on material cost will be influenced by the manufacturing process and the materials employed. To ensure the manufacturing of high-quality products at an affordable price, manufacturers must carefully balance the necessary level of precision with the associated material costs.

Based on a comprehensive review of existing literature, it is evident that there is a significant gap in research on PEMFCs, particularly in addressing how dimensional uncertainties in design variables affect $V_{cell}$, while concurrently aiming to reduce material costs during manufacturing. In this study, an RBDO approach has been introduced to PEMFC design, which accounts for manufacturing uncertainties and enhances reliability. The four key PEMFC design variables, gas diffusion layer thickness ($\delta_{gdl}$), channel depth ($d_{ch}$), channel width ($w_{ch}$) and land width ($w_{land}$), have been considered for optimization with the objective of maximizing $V_{cell}$ while considering the material cost of the cathode gas diffusion layer ($Cost_{cGDL}$) and cathode bipolar plate $\left(Cost_{cBP}\right)$. At first, under a suitable constraint condition, initial data samples for the optimization study are generated using a Latin hypercube sampling (LHS) technique. These data samples are then considered as inputs for building a database of $V_{cell}$ values through CFD simulations of a comprehensive, multi-scale, two-phase, 3D numerical PEMFC model, which has been extensively developed and experimentally validated in our previous studies [29,30,31,32]. Further, the database of design variables and their corresponding $V_{cell}$ values are divided into training and test sample sets. Considering the training samples, a multi-layer perceptron (MLP) surrogate model is constructed using MATLAB R2024a, and then tested on distinct unseen test samples. Next, the MLP is linked to a particle swarm optimization (PSO) algorithm and a constrained DDO is performed focusing on maximizing $V_{cell}$. To address manufacturing uncertainties, we introduce the RBDO technique, which differs from the current DDO method by centering this study on meeting practical engineering reliability norms of 95%. Furthermore, to evaluate the effects of uncertainty and present an unfailing design, the reliability of the two constraints $Cost_{cGDL}$ and $Cost_{cBP}$ of a 100 kW road vehicle stack has been assessed and presented. The RBDO approach effectively balances $V_{cell}$ and reliability, achieving a target reliability of 95% for both $Cost_{cGDL}$ and $Cost_{cBP}$. This strategy has significant implications for the deployment of PEMFC technology in automobiles, demonstrating the potential for widespread adoption and environmental impact.

## 2. Numerical PEMFC Model

In this optimization study, a 3D, multiscale, two phase PEMFC model has been used. The model is based on the multiphase mixture (M^2^) model proposed by Wang and Cheng [33] and considers various components of a PEMFC cell which includes the BPs, GDLs, CLs and the membrane. The model has been validated against the experimental polarization curves measured under different cell designs and operating conditions [30,34]. For a realistic model, the effects of clamping on the GDL structure and the variation in properties have been considered. Since the model employed in this study is identical to that described in our previous studies [30,35,36], the model assumptions and governing equations are presented in brief in Section 2.1 and Section 2.2. Finally, in Section 2.3, an outline of the boundary conditions and numerical implementation of the PEMFC model using ANSYS fluent (ANSYS Inc., Canonsburg, PA, USA) has been presented. 

### 2.1. Model Assumptions

The following are the specific assumption used in this numerical study:

1. The operating pressure is low, and hence, ideal gas mixtures are assumed in the gas phase.
2. The velocity of flow is low and laminar.
3. The effect of gravity is neglected.
4. In the porous region, immobile liquid saturation is neglected.

### 2.2. Governing Equations and Source Terms

In this study, the PEMFC model under consideration is governed by the five conservation equations: mass, momentum, species, charge, and thermal energy. The equations stated above are linked to source terms that are related to the hydrogen oxidation reaction (HOR) in the anode and oxygen reduction reaction (ORR) in the cathode. For further reference regarding the governing equations and source terms, readers can refer Table 1 and Table 2.

**Table 1:** Governing equations for the PEMFC model.

Governing Equations		
Mass	$\nabla \cdot \left(\rho \vec{u}\right)=0$	(1)
Momentum	$\left(\frac{1}{\varepsilon^{2}}\right)\nabla \cdot \left(\rho \vec{u}\vec{u}\right)=-\nabla P+\nabla \cdot \tau +S_{u}$	(2)
Species	Flow channels and porous media: $\nabla \cdot \left(\gamma_{i}\rho m_{i}\vec{u}\right)=\nabla \cdot \left[\rho^{g}D_{i}^{g,eff}\nabla \left(m_{i}^{g}\right)\right]+\nabla \cdot \left[\left(m_{i}^{g}-m_{i}^{l}\right){\vec{j}}^{l}\right]+S_{i}$	(3)
	Water transport in membrane: $\nabla \cdot \left(\left(\frac{\rho^{mem}}{EW}\right)D_{w}^{mem}\nabla \lambda \right)M_{w}-\nabla \cdot \left(n_{d}\left(\frac{I}{F}\right)\right)M_{w}+\nabla \cdot (\left(\frac{\kappa^{mem}}{\nu^{l}}\right)\nabla P^{l}=0$	(4)
Charge	Proton transport: $\nabla \cdot \left(\kappa^{eff}\nabla \phi_{e}\right)+S_{\phi }=0$	(5)
	Electron transport: $\nabla \cdot \left(\sigma^{eff}\nabla \phi_{s}\right)-S_{\phi }=0$	(6)
Energy	$\nabla \cdot \left(\rho \vec{u}C_{p}^{g}T\right)=\nabla \cdot \left(k^{eff}\nabla T\right)+S_{T}$	(7)

**Table 2:** Source/sink terms and physicochemical relations used in the PEMFC model.

Description	Expressions	
**Momentum**		
Porous media	$S_{u}=-\frac{\mu }{K}\vec{u}$	
**Species**		
H_2_ in anode CL	$S_{H_{2},a}=\left[-\frac{j}{2F}\right]M_{H_{2}}$	
O_2_ in cathode CL	$S_{O_{2},c}=\left[\frac{j}{4F}\right]M_{O_{2}}$	
Water in anode CL	$S_{w,a}=\left[-\nabla \cdot \left(\frac{n_{d}}{F}\vec{I}\right)+\frac{j}{4F}\right]M_{w}$	
Water in cathode CL	$S_{w,c}=\left[-\nabla \cdot \left(\frac{n_{d}}{F}\vec{I}\right)-\frac{j}{2F}\right]M_{w}$	
**Energy**		
In anode CL	$S_{T,a}=j\cdot \eta +\frac{I^{2}}{k^{eff}}$	
In cathode CL	$S_{T,c}=j\left(\eta +T\frac{dU_{0}}{dT}\right)+\frac{I^{2}}{k^{eff}}$	
In membrane	$S_{T}=\frac{I^{2}}{k^{eff}}$	
**Charge**		
In CLs	$S_{\phi }=j$	
**Electrochemical reactions**		
General form	$\sum_{k}s_{i}M_{i}^{z}=ne^{-},where\left\{\begin{array}{c} M_{i}=chemical\;formula\;of\;species\;i\\ s_{i}=stoichiometric\;coefficient\\ n=number\;of\;electrons\;transferred \end{array}\right.$	
In the anode side (HOR)	$H_{2}-2H^{+}=2e^{-}$	
In the cathode side (ORR)	$2H_{2}O-O_{2}-4H^{+}=4e^{-}$	
**Transfer current density,** $\left[A/m^{3}\right]$		
In anode CL (HOR)	$j=\left(1-s\right)ai_{0,a}^{ref}{\left(\frac{C_{H_{2}}}{C_{H_{2},ref}}\right)}^{\frac{1}{2}}\exp\left[-\frac{E_{a}}{R}\left(\frac{1}{T}-\frac{1}{353.15}\right)\right]\left(\exp\left(\frac{\alpha_{a}F}{RT}\eta \right)-\exp\left(-\frac{\alpha_{c}F}{RT}\eta \right)\right)$	(8)
In cathode CL (ORR)	$j=-\frac{3L_{Pt}}{{r_{Pt}{\cdot \rho }_{Pt}\cdot \delta }_{CL}}i_{0,c}^{ref}{\left(\frac{C_{O_{2}}^{Pt}}{C_{O_{2},ref}}\right)}^{3/4}exp\left(-\frac{E_{c}}{R}\left(\frac{1}{T}-\frac{1}{353.15}\right)\right)\exp\left(-\frac{\alpha_{c}}{R_{u}T}F\eta \right)$	(9)
**Overpotential**		
General form	$\eta =\phi_{s}-\phi_{e}-U$ where, $U=U_{0}-\frac{RT}{nF}\ln\frac{C_{O_{2}}}{C_{O_{2\;ref}}}$	(10)

In Table 3, the kinetic, transport and physiochemical properties of the PEMFC components have been listed. Table 4 lists all of the pertinent equations that relate to the two-phase mixture model suggested by Wang and Cheng [33]. Additionally, Table 5 lists a set of species transport properties which are correlated to the water content λ, which in turn is a function of the water activity a [37]. The key factor behind developing the two-phase mixture model is to predict flooding and its impact on the performance of the fuel cell. In Table 4, Equations (13)–(27) are the expressions used in the two-phase mixture model that aid in capturing the dynamics of liquid water formation and distribution within the cell. Additionally, the Butler–Volmer equation, Equations (8) and (9) in Table 2, reflects the impact of liquid saturation (1-*s*) on the reaction kinetics, providing a detailed understanding of how water content affects the electrochemical reactions. The multiscale aspect of the model focuses on the transport phenomena of oxygen through the catalyst particle, encompassing the water film, ionomer film and Pt particle at a micro scale. For further insights, please refer to Vaz et. al. [29], which provides details regarding the microscale transport losses, highlighting the resistances encountered by oxygen molecules as they navigate through these different layers. By incorporating these detailed mechanisms, the model offers a comprehensive view of the factors influencing PEMFC performance, from macroscopic flooding effects to microscopic transport losses. This multiscale, two-phase approach allows for a more accurate prediction and optimization of fuel cell behavior under various operating conditions.

**Table 3:** Kinetic, transport and physiochemical properties.

Description	Value/Expression	
Exchange current density of HOR × ECSA per unit CL volume, ${aI}_{0,a}^{ref}$	1.2 × 10^10^ A/m^3^	[34]
Exchange current density for the ORR, $I_{0,c}^{ref}$	2.0 × 10^−4^ A/cm^2^-Pt	[34]
Activation energy of the anode, $E_{a}$	10.0 kJ/mol	[38]
Activation energy of the cathode, $E_{c}$	70.0 kJ/mol	[39]
Transfer coefficient of the HOR, $\alpha_{a}=\alpha_{c}$	1	[40]
Transfer coefficient of the ORR, $\alpha_{c}$	1	[40]
Reference H_2_/O_2_ molar concentration, $C^{ref}$	40.88 mol/m^3^	[34]
Permeability of the GDL, $K_{GDL}$	1.0 × 10^−12^ m^2^	[41]
Permeability of the CL, $K_{CL}$	1.0 × 10^−13^ m^2^	[41]
Porosity of the GDL, $\varepsilon_{GDL}\;$	0.64	[42]
Porosity of the CL, $\varepsilon_{CL}$	0.4	[43]
Equivalent weight of electrolyte in the membrane, EW	1.1 kg/mol	[44]
Youngs modulus of the GDL	6.16MPa	[45]
Poisson ratio of the GDL	0.09	[36]
Faraday’s constant, F	96,485 C/mol	
Universal gas constant, $R_{u}$	8.314 $J/\left(mol\cdot K\right)$	
H_2_ diffusivity in the anode gas channel, $D_{0,H_{2},a}^{g}$	1.1028 × 10^−4^ m^2^/s	[46]
H_2_O diffusivity in the anode gas channel, $D_{0H_{2}O,a}^{g}$	1.1028 × 10^−4^ m^2^/s	[46]
O_2_ diffusivity in the cathode gas channel, $D_{0,O_{2},c}^{g}$	3.2348 × 10^−4^ m^2^/s	[46]
H_2_O diffusivity in the cathode gas channel, $D_{0,H_{2}O,c}^{g}$	7.35 × 10^−5^ m^2^/s	[46]
Multi component gas diffusivity ($D_{i}^{g})$	For nonporous regions $D_{i}^{g}=\frac{1-x_{i}\;}{\sum_{\begin{array}{c} j\\ j\neq 1 \end{array}}^{j=n}\frac{x_{j}}{D_{i,j}}}$	[47]
	where, $D_{i,j}=\frac{1.013\times 10^{-7}\times T^{1.75}}{p\;.\;{\left(\chi_{i}^{\frac{1}{3}}+\chi_{j}^{\frac{1}{3}}\right)}^{2}}$ . ${\left(\frac{1}{M_{i}}+\frac{1}{M_{j}}\right)}^{1/2}$ where, $\chi_{H_{2}}=7.07,\;\chi_{w}=12.7,\;\chi_{N_{2}}=17.9,\;\chi_{O_{2}}=16.6$	(11)
Effective diffusivity ($D_{i}^{g,eff})$	For porous regions $D_{i}^{g,eff}=\varepsilon^{\tau }\cdot D_{i}^{g}$	(12)

**Table 4:** Expressions used in the two-phase mixture model.

Description	Expression	
Mixture density	$\rho =\rho^{l}s+\rho^{g}\left(1-s\right)$	(13)
Gas mixture density	$\rho^{g}=\left(\frac{P}{R_{u}T}\right)\frac{1}{\sum_{i}\frac{m_{i}^{g}}{M_{i}}}$	(14)
Mixture velocity	$\rho \vec{u}=\rho^{l}{\vec{u}}^{l}+\rho^{g}{\vec{u}}^{g}$	(15)
Mixture mass fraction	$m_{i}=\frac{\rho^{l}sm_{i}^{l}+\rho^{g}\left(1-s\right)m_{i}^{g}}{\rho }$	(16)
Relative permeability	$k_{r}^{l}=s^{3}$	(17)
	$k_{r}^{g}=(1-s{)}^{3}$	(18)
Kinematic viscosity of the two-phase mixture	$v={\left(\frac{k_{r}^{l}}{v^{l}}+\frac{k_{r}^{g}}{v^{g}}\right)}^{-1}$	(19)
Kinematic viscosity of the gas mixture	$v^{g}=\frac{\mu^{g}}{\rho^{g}}=\frac{1}{\rho^{g}}\sum_{i=1}^{n}\frac{x_{i}\mu_{i}}{\sum_{j=1}^{n}x_{j}\phi_{ij}}$	(20)
	where $\phi_{ij}=\frac{1}{\sqrt{8}}{\left(1+\frac{M_{i}}{M_{j}}\right)}^{-1/2}{\left[1+{\left(\frac{\mu_{i}}{\mu_{j}}\right)}^{1/2}{\left(\frac{M_{j}}{M_{i}}\right)}^{1/4}\right]}^{2}$	(21)
	and $\mu_{i}\left[N.s.m^{-2}\right]=\left\{\begin{array}{c} \mu_{H_{2}}=0.21\times 10^{-6}T^{0.66}\\ \mu_{w}=0.00584\times 10^{-6}T^{1.29}\\ \mu_{N_{2}}=0.237\times 10^{-6}T^{0.76}\\ \mu_{O_{2}}=0.246\times 10^{-6}T^{0.78} \end{array}\right.$, T in kelvin	(22)
Relative mobility	$\lambda^{l}=\frac{k_{r}^{l}}{v^{l}}v$	(23)
	$\lambda^{g}=1-\lambda^{l}$	(24)
Diffusive mass flux	$\vec{j^{l}}=\rho^{l}{\vec{u}}^{l}-\lambda^{l}\rho \vec{u}=\frac{K}{v}\lambda^{l}\lambda^{g}\nabla P_{c}$	(25)
Capillary pressure *P_c_*	$P_{c}=P^{g}-P^{l}=\sigma \cos\theta {\left(\frac{\varepsilon }{K}\right)}^{1/2}J\left(s\right)$	(26)
Leverett function *J*(*s*)	$J=\left\{\begin{array}{c} 1.417(1-s)-2.120(1-s{)}^{2}+1.263(1-s{)}^{3}\\ 1.417s-2.120s^{2}+1.263s^{3} \end{array}\right.\begin{array}{c} \mathrm{if}{\;\theta }_{c}<90^{\circ }\\ \mathrm{if}{\;\theta }_{c}>90^{\circ } \end{array}$	(27)

**Table 5:** Transport properties in the electrolyte.

Description	Expression	
Water activity	$a=\frac{C_{w}^{g}R_{u}T}{P_{sat}}$	(28)
Membrane water content	$\lambda =\left\{\begin{array}{c} \lambda_{g}=0.043+17.81a-39.85a^{2}+36.0a^{3}\;for\;0<a\leq 1\\ \lambda_{l}=22\; \end{array}\right.$	(29)
Electro-osmotic drag (EOD) coefficient of water	$n_{d}=\frac{2.5\lambda }{22}$	(30)
Proton conductivity	$\kappa =\left(0.5139\lambda -0.326\right)\exp\left.\left[1268\left(\frac{1}{303}-\frac{1}{T}\right)\right.\right]$	(31)
Water diffusion coefficient	$D_{w}^{mem}=\left\{\begin{array}{c} 2.692661843\cdot 10^{-10}\;for\;\lambda \leq 2\\ \left\{0.87\left(3-\lambda \right)+2.95(\lambda -2)\}\cdot 10^{-10}\cdot e^{\left(7.9728-2416/T\right)}\;for\;2<\lambda \leq 3\right.\\ \left\{2.95\left(4-\lambda \right)+1.642454\left(\lambda -3\right)\right\}\cdot 10^{-10}\cdot e^{\left(7.9728-2416/T\right)}\;for\;3<\lambda \leq 4\\ \left(2.563-0.33\lambda +0.0264\lambda^{2}-0.000671\lambda^{3}\right)\cdot 10^{-10}\cdot e^{\left(79728-2416/T\right)}\;for\;4<\lambda \leq \lambda_{a=1}^{g} \end{array}\right.$	(32)
Interfacial resistance of the water film	$\Omega_{w,int}=z_{w}\frac{\delta_{w}}{D_{O_{2},w}}$	(33)

### 2.3. Boundary Conditions and Numerical Implementation

**Figure 1:**
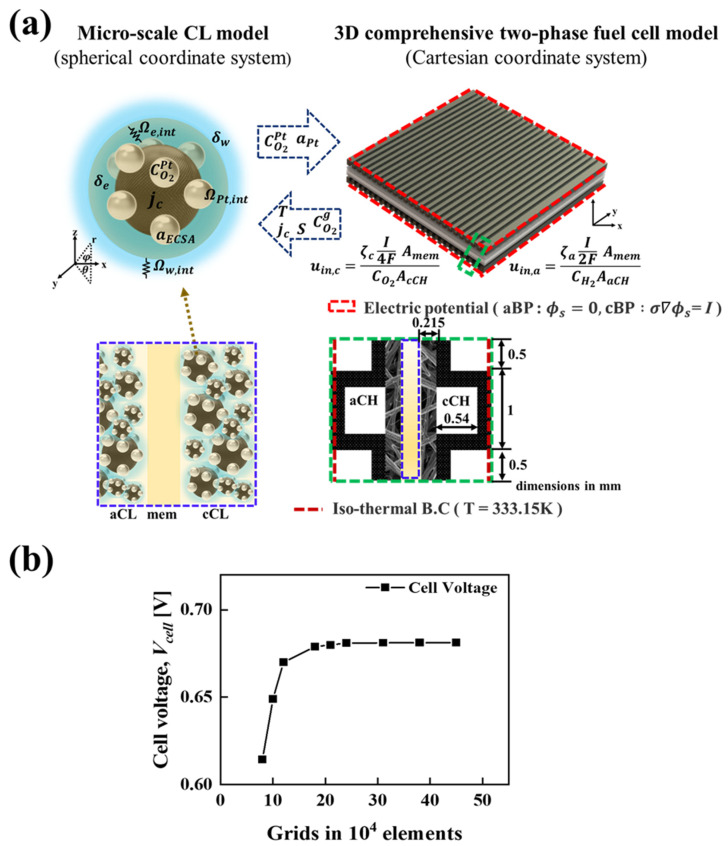
(**a**) Illustration of the micro- and macro-scale computational domains in PEMFCs, emphasizing the variables exchanged during 3D multi-scale simulations. (**b**) Grid independence test results, demonstrating the stability and accuracy of the computational model across various grid resolutions.

Figure 1 illustrates the micro and macro scale computational domains of the PEMFC along with the various switching variables exchanged during the 3D multi-scale simulations. The figure includes the structure of an individual unit cell and boundary conditions considered in the present study. Apart from the inlet and outlet regions of the anode and cathode gas channels, all of the external surfaces have been considered for mass flow under the no-slip and impermeability boundary conditions. In terms of thermal boundary conditions in the computational domain, an isothermal boundary condition is considered for the side walls of the anode and cathode, whereas an adiabatic boundary condition is considered for the top and bottom surfaces. The PEMFC can be operated either in the galvanostatic or potentiostatic mode and this can be achieved by applying a constant voltage or current density at the outer side wall of the cathode, while the electric potential $\phi_{s}$ is fixed to zero. Figure 1b presents the results of the grid-independent study and the number of meshes required to achieve good analysis accuracy is determined to be about 240,000. The PEMFC model considered in this study is numerically implemented by employing user-defined functions in the commercially available CFD program ANSYS Fluent ver. 23 (ANSYS, Inc., Canonsburg, PA, USA) and the convergence criteria is set to 10^−8^ for the equation residuals.

## 3. Overview of Design Optimization Strategies for Engineering Applications

In engineering design, the two fundamental strategies typically employed to optimize a design are the DDO and RBDO approach. The DDO method is centered around maximizing or minimizing a single/multi-objective function while adhering to a specific set of constraints. This strategy is widely followed in industries and is computationally efficient and provides a well-defined single design solution. However, the RBDO approach considers the uncertainties in design variables and aims to achieve a set level of reliability. This method involves estimating the probability of failure under different uncertain conditions, and using this information, the design variables are estimated to minimize the probability of failure. When compared to the DDO approach, RBDO is a more sophisticated design strategy as it considers the inherent variability and uncertainties associated with the design variables. 

A typical DDO problem for a single-objective optimization problem with the objective of minimizing the cost function f(X) is formulated as

(34) Minimize f(X)

subjected to

$$G_{j}\left(X\right)\geq 0\;for\;j=1,\ldots ,J$$

(35) $$X_{i}^{L}\leq X\leq \;X_{i}^{U},\;for\;i=1,\ldots ,N$$

where $X={\left\{\;X_{1},\;X_{2},\;\ldots ,\;X_{N}\right\}}^{T}$ is the vector of N input design variables, and J is the number of constraints. $G_{j}\left(X\right)\geq 0\;$ represents the inequality constraint where all of the constraints J are satisfied. The constraints are violated if $G_{j}\left(X\right)<0$. The terms $X_{i}^{U}$ and $X_{i}^{L}$ represent the lower and upper bounds of the N design variables.

**Figure 2:**
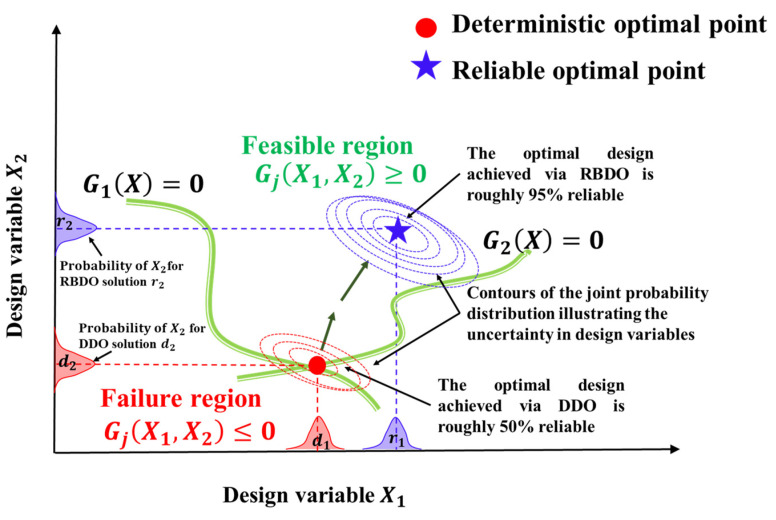
Illustrations depicting the outcomes of RBDO and DDO on the solution of a hypothetical optimization problem, highlighting the impact of uncertainty in the design variables $X_{1}$ and $X_{2}$.

Figure 2 shows the solution for a deterministic single-objective optimization problem with two design variables ($X_{1},X_{2}$), and two constraints, $G_{1}(X)$ and $G_{2}\left(X\right),\;$ obtained through a DDO approach such that Equation (34) is satisfied. As seen, the deterministic optimum point denoted by $X_{1}=d_{1},\;X_{2}=d_{2}$ lies at the intersection of the two constraint curves denoted by $G_{1}\left(X\right)=0$ and $G_{2}\left(X\right)=0$. The region below the constraint boundaries is known as the infeasible region. The solution for Equations (34)–(35) is said to be violated when the constraint solution is $G_{j}\left(X_{1},X_{2}\right)\leq 0$ and is said to be acceptable when $G_{j}\left(X_{1},X_{2}\right)\geq 0$. The region dividing the feasible and infeasible regions denoted is denoted by the constraint boundaries, $G_{1}\left(X\right)=0$ and $G_{2}\left(X\right)=0$. When optimization is performed using the DDO approach without accounting for uncertainties in the design variables, there is a significant risk that the optimal design will exceed the constraint limits, potentially resulting in the failure of the DDO design.

The basic idea underlying RBDO is to apply a numerical optimization technique to ensure that the optimal design meets the reliability criteria under uncertainty. The RBDO problem is formulated to balance performance and reliability, incorporating probabilistic constraints to account for uncertainties in design parameters. A general RBDO problem is further formulated as follows:

(36) Minimize f(d)

subjected to

$$P_{Fj}\left(d\right)=P\left[G_{j}\left(X\right)>0\right]\leq P_{Fj}^{tar}\;\mathrm{for}\;j=1,\ldots ,J$$

(37) $$d_{i}^{L}\leq d\leq d_{i}^{U}\;for\;i=1,\ldots ,N$$

where $d={\left\{\;d_{1},\;d_{2},\;\ldots ,\;d_{N}\right\}}^{T}$ is the vector of N random input design variables which is made up of the mean values for each of the N random design variables. d can be further represented as d=μ(X), where μ(•) is the mean value operator and $X={\left\{\;X_{1},\;X_{2},\;\ldots ,\;X_{N}\right\}}^{T}$ is the random design variable vector. $P_{Fj}\left(d\right)$ represents the probability of failure at the jth constraint of the design vector d, $G_{j}$ represents the jth constraint, $P\left[\cdot \right]$ is the probability that the jth  constraint is violated, J is the number of constraints, $P_{Fj}^{tar}$ is the target probability of failure for the jth  constraint and $d_{i}^{L}$ and $d_{i}^{U}$ are the lower and upper bounds of the random input design variables in the design space. 

The RBDO solution to Equations (36) and (37) is shown in Figure 2, where it can be compared to the DDO problem’s solution. The reliable solution, as indicated by the blue star, is in the feasible region and has a slightly higher objective functional value than the deterministic design, ensuring compliance with reliability constraints. The deterministic optimal point, initially in the failure region (red circle), is adjusted in RBDO to meet a target probability of failure, ensuring the design remains within the feasible region. For instance, at the DDO optimal, 50% of the joint probability contours exceed the constraint boundary, reflecting a 50% reliability. Conversely, the RBDO method achieves a reliable optimal with 95% reliability, as only 5% of the total violates the constraint condition.

**Figure 3:**
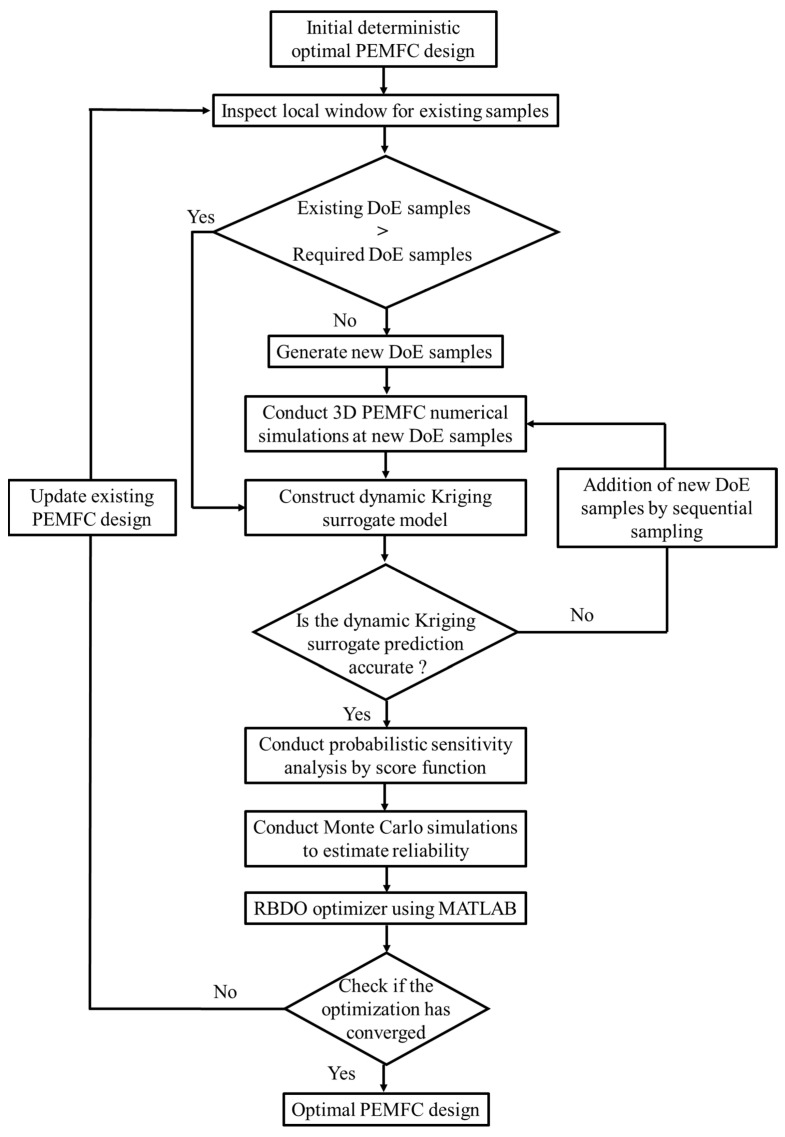
Flowchart of the RBDO process beginning with the initial deterministic PEMFC design and sequentially following through with scanning for Design of Experiments (DoE) samples, generating additional samples if needed, executing 3D PEMFC simulations, constructing and verifying the dynamic Kriging surrogate model and performing Monte Carlo simulations for reliability assessment. The process concludes with the RBDO optimizer determining if an optimal design convergence has been achieved.

Figure 3 demonstrates the process flow for estimating a reliable optimal design solution using design sensitivity- and sampling-based RBDO with Reliability Analysis & Multidisciplinary Design Optimization (RAMDO) 2022.1 software. As seen, the RBDO process starts with a deterministic optimal design. In this study, we attain the deterministic optimal point using the PSO algorithm in conjunction with the MLP surrogate model, as detailed in our previous research. For a comprehensive description of the model and optimization algorithm, interested readers can refer to our earlier studies [29,48]. Further, at the deterministic optimal point, the input design variables, i.e., $\delta_{gdl}$, $d_{ch}$, $w_{ch}$ and $w_{land}$ are assumed to follow a marginal normal input distribution. These input distributions are further used to construct the dynamic Kriging (DKG) surrogate model which is basically an approximation of the true PEMFC numerical model. The construction of the DKG surrogate is based on the Design of Experiments (DoE) where a combination of input parameters from the respective normal distributions, and the 3D PEMFC simulation model, are used to generate the performance of the PEMFC. Once the surrogate is constructed, the accuracy of the surrogate must be verified. The mean square error (MSE) is used as a metric for checking the accuracy of the constructed surrogate and is set to 0.001. The surrogates developed are then utilized to direct reliability estimation via Monte Carlo simulation (MCS). Given that MCS requires very large sample points, evaluations of true samples make it practically impossible. As a result, the use of the DKG surrogate makes the task easier. The entire optimization scheme is based on a single loop optimization structure and has a significant reduction in computational time when compared to a reliability-based index approach and the performance measure approach. 

## 4. Estimating the Material Costs of the Cathode GDL and BP in PEMFCs

The fuel cell system’s acquisition cost needs to be reduced to a level comparable with that of an internal combustion engine for PEMFCs to be a viable choice for commercial application. A study by Simon et al. [49] reveals that the fuel cell stack accounts to approximately 45% of the total system cost, while the majority of the cost is contributed by the MEAs (includes catalysts, membrane, GDLs and the MEA assembly) and the BPs. At high production volumes of above 500,000 stacks/year, the materials used in the GDL and BP dominate most of the manufacturing expenses, accounting for 89% and 57% of the total production cost incurred [50]. Thus, estimating the material cost of the GDL and BP in PEMFCs is crucial to ensure the economic viability of this clean energy technology. In addition, an accurate estimation of material costs enables manufacturers to plan budgets effectively and help optimize the production process, thereby reducing the overall costs. 

In this study, we base our cathode GDL material cost estimation model on Ballard material products, which are comparable to those from other GDL manufacturers [51]. The production of a GDL involves two main steps, carbon fiber papermaking and hydrophobic treatment. First, carbon fibers are chopped and mixed with water and polyvinyl alcohol. This mixture is then laid onto a web using a wet-laid papermaking technique, dried and re-spooled. To control the porosity, the carbon and resin content is carefully regulated, followed by heat treatment under oxidation conditions. Finally, fluorinated ethylene propylene is added to the surface to enhance hydrophobicity. As reported by Brian et al. [51], the $Cost_{cGDL}$ of a GDL with a thickness of 105 microns is 1.58$\;\$/m^{2}$. This reference material cost includes various components such as paper making, impregnation coating for porosity, oxidation/carbonization/graphitization and impregnation coating for hydrophobicity. By summing up the individual material cost at each step involved, we derive the following single equation for the total material cost of a GDL:

(38) $$Cost_{cGDL}=A_{cell}\times N_{cell}\times \frac{\delta_{gdl}}{\delta_{ref}}\times Cost_{GDL}^{ref}$$

where $A_{cell}$ represents the active area of the cell, set at 0.03 $m^{2}$, $N_{cell}$ is the number of cells needed to produce a stack of 100 kW, set at 550, $\delta_{ref}$ is the thickness of the reference GDL, set at 105 micron, and $Cost_{GDL}^{ref}$ is the material cost of the reference GDL, set at 1.58$\;\$/m^{2}$.

Further, to estimate the material cost of the cathode BP, the material cost equation reported by the Battelle Memorial Institute [52] has been used and is given as follows:

(39) $$m_{cBP}=\rho_{BP}\times A_{cell}\times {(2d}_{ch}+d_{BP})\times O_{allw}$$

(40) $$Cost_{cBP}=m_{BP}\times N_{cell}\times Cost_{BP}^{mat}$$

where $m_{cBP}$ is the mass of the cathode BP, $\rho_{BP}$ is the density of the BP material used and is considered as 1900$\;kg/m^{3}$, the term $d_{BP}$ represents the thickness of the base material in the BP, set at 1 mm, $O_{allw}$ is the overage allowance and is considered as 5%, and $Cost_{BP}^{mat}$ represents the cost of the bipolar material used and is given as 2.066 $/kg.

## 5. Formulation of the Optimization Problem

The modern engineering community is increasingly using optimization as a design tool to achieve optimal designs that minimize costs while meeting performance constraints. Optimization is used to find optimal designs characterized by lower costs while satisfying performance constraints. In the present study, two design optimization strategies for PEMFCs, DDO and RBDO, have been used. In DDO, the primary objective is to maximize the performance of the objective function $\left(V_{cell}\right)$, while adhering to specific deterministic constraints and design variable bounds. This approach ensures that the design variables are optimized within predefined bounds, leading to a solution that meets the set criteria without considering uncertainties. Considering the aforementioned criteria for DDO, the optimization problem is formulated as follows:

(41) $$\mathrm{Maximize}\;V_{cell}(X)$$

$$subject\;to\left\{\begin{array}{c} Cost_{cGDL}\left(X\right)\geq 0\;\\ Cost_{cBP}\left(X\right)\geq 0 \end{array}\right.\;\mathrm{for}\;X^{L}\leq X\leq X^{U}$$

$$X^{L}=[0.05,0.3,0.3,0.3]$$

(42) $$X^{U}=[0.4,2,2,1]$$

where X is the vector of the four design variables represented as $X={\left\{\delta_{gdl},\;d_{ch},\;w_{ch},\;w_{land}\right\}}^{T}$, and $X^{L}$ and $X^{U}$ represent the lower and upper bounds of the design variables. The deterministic optimization described in Equations (41) and (42) does not account for uncertainties in the design variables. As a result, the optimized designs obtained through DDO have a high probability of failure due to violations of the constraints which would lead to potential design failures. 

In a fuel cell system, the material employed has a significant impact on its performance, reliability and cost. As previously discussed, the material costs on the cathode side, particularly for the GDL and BP, are significant throughout the manufacturing process. The permissible variation in the dimensions of the GDLs and BP, known as tolerance, is critical during production. Therefore, it is essential to study the consequences of tolerance variations to mitigate their impact on material costs. To address these challenges, RBDO is employed. In RBDO, material costs for BP and GDL are considered as reliability constraints, ensuring that the design remains robust under uncertainty. The problem can be formulated as follows:

(43) $$Maximize\;V_{cell}\left(d\right)$$

(44) $$subject\;to\left\{\begin{array}{c} P_{F1}\left(d\right)=P\left[Cost_{cGDL}(X)\leq Cost_{cGDL}^{DDO}\right]\leq P_{F1}^{tar}\\ P_{F2}\left(d\right)=P\left[Cost_{cBP}(X)\geq Cost_{cBP}^{DDO}\right]\leq P_{F2}^{tar} \end{array}\right.$$

where d represents the design vector and $d={\left\{\mu \left(X_{\delta_{gdl}}\right),\mu \left(X_{d_{ch}}\right),\mu \left(X_{w_{ch}}\right),\mu \left(X_{w_{land}}\right)\right\}}^{T}$, which is the mean value of the four design variables, $\delta_{gdl},\;d_{ch},\;w_{ch}$ and $w_{land}$. The terms $P_{F1}\left(d\right)\;$ and $P_{F2}\left(d\right)$ are defined as the probability of failure of the two constraint function vector at the design vector d and X is the random design variable vector represented as $X=\{X_{\delta_{gdl}},\;X_{d_{ch}},\;X_{w_{ch}},\;X_{w_{land}}\}$. The two constraints, $Cost_{cGDL}^{DDO}$ and $Cost_{cBP}^{DDO}$ represent the material cost of the cathode GDL and BP at the deterministic optimal as a result of the DDO. The target probability of failure $P_{F1}^{tar}$ and $P_{F2}^{tar}$ for the two constraints, $Cost_{cGDL}$ and $Cost_{cBP}$ is considered as 5%, where the target reliability is 95%.

## 6. Results and Discussion

In this study, the results of an optimization framework aimed towards optimizing fuel cell stack performance while taking into account the cathode, GDL and BP material costs as constraints have been presented. The analysis is carried out with the $V_{cell}$ as the objective function that is being maximized, while the dimensional uncertainties of the four key PEMFC design variables, i.e., $\delta_{gdl},\;$ $d_{ch},\;w_{ch}$ and $w_{land},$ have been considered. In addition, the cost of the materials for the cathode, GDL and BP is also considered as a criterion that must be met. In Section 6.1, we evaluate the predictive capability of the MLP surrogate. In Section 6.2, the results of the DDO approach are presented. Furthermore, in Section 6.3, a comprehensive discussion of the RBDO approach wherein the uncertainties in design variables have been considered has been presented. 

**Figure 4:**
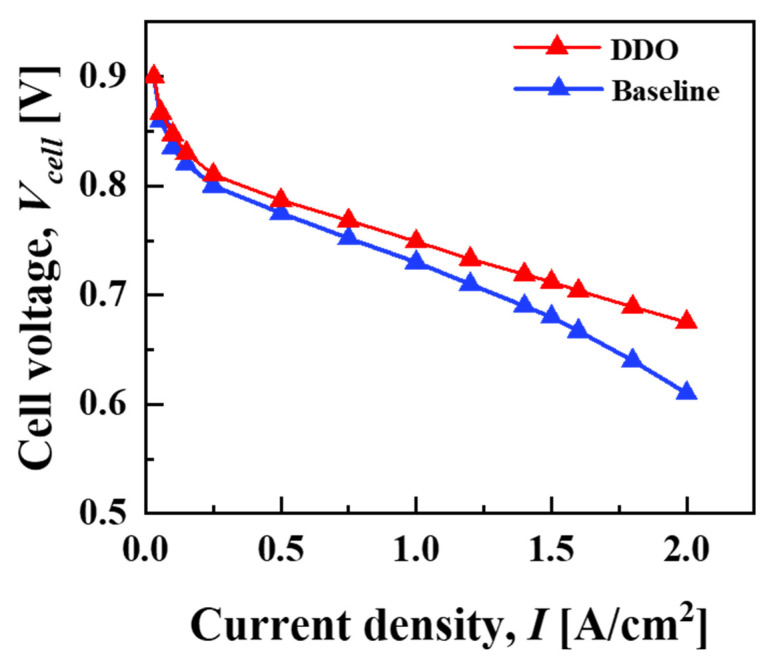
Comparison of simulated polarization curves for the baseline and deterministic optimal design predicted with DDO strategy via MLP–PSO surrogate. The simulations are conducted under controlled operating conditions for the anode and cathode, with both set at pressures of 2 bar and stoichiometries of 1.2 and 2.0, respectively, and with inlet relative humidity maintained at 100% for each. The cell is operated at an operating temperature of 333.15K. The baseline design is with $\delta_{gdl}=0.215\;mm,\;d_{ch}=0.54\;mm,w_{ch}=1\;mm\;$ and $w_{land}=1\;mm$ and the deterministic optimal design is with $\delta_{gdl}=0.188\;mm,\;d_{ch}=0.3\;mm,\;w_{ch}=0.614\;mm\;$ and $w_{land}=0.310\;mm$.

As seen in Figure 4, to ensure that the 3D numerical PEMFC model considered in the present study can predict the performance of the fuel cell, a 3D PEMFC numerical model that was previously developed and validated against experimental polarization curves under various cell designs and operating conditions has been taken into consideration [31]. This validated model has been considered as the baseline for the present optimization study and has incorporated a $\delta_{gdl}=0.215\;mm,\;d_{ch}=0.54\;mm$, $\;w_{ch}=1\;mm$ and $w_{land}=1\;mm$, under 20% GDL compression (the thickness of GDL is reduced by 20% from its initial value). These values are consistent and well defined within the range of the design variable space specified in Equations (41) and (42). 

### 6.1. Evaluation of Predictive Capability of the MLP Surrogate

To estimate the maximum $V_{cell}$ via surrogate-based design optimization under suitable constraint conditions as described in Section 5, at first, an MLP surrogate model that was developed using MATLAB, with detailed construction and implementation described in our previous work [29,48], was utilized. The model was trained with a dataset of 75 samples. To effectively evaluate the predictive performance of the trained MLP model, a separate test set comprising 15 samples, distinct from the trained set, was employed. An error analysis was conducted to measure the predictive capability of the trained surrogate, using root mean square error (RMSE) and $adjusted\;R^{2}$ error metrices, which are described in Equations (45) and (46), respectively.

(45) $$RMSE=\sqrt{\frac{1}{N}{\sum_{i=1}^{N}{(\hat{v}}_{i}-v_{i})}^{2}}$$

(46) $$adjusted\;R^{2}=1-\frac{N-1}{N-d-1}\left(1-\left(1-\frac{\sum_{i=1}^{N}{\left(v_{i}-{\hat{v}}_{i}\right)}^{2}}{\sum_{i=1}^{N}{\left(v_{i}-{\bar{v}}_{i}\right)}^{2}}\right)\right)$$

where $v_{i}$ and ${\hat{v}}_{i}$ denote the responses of the 3D PEMFC simulations and the predicted values of the MLP model, respectively, ${\bar{v}}_{i}$ denotes the mean value of the observed data at the N test points and *d* is the number of design variables. RMSE measures the average magnitude of the errors between the predicted and actual values. Lower RMSE values indicate better model performance, with values approaching zero indicating the best accuracy. The $adjusted\;R^{2}$ value ranges from zero to one; a value of zero indicates that the model does not explain any of the variability in the response data around its mean, and a value of one indicates that the model is capable of considering the variability in the response data around its mean. 

**Figure 5:**
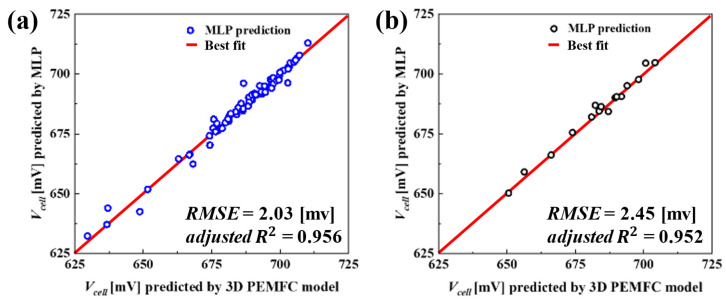
Scatter plots illustrating the prediction accuracy of the MLP surrogate for (**a**) training data and (**b**) test data. The plots highlight the correlation between predicted and actual values, with performance metrics RMSE and adjusted R^2^ indicated for each dataset.

Figure 5 illustrates a scatter plot comparing the prediction capability of the MLP surrogate for both the training and test datasets. As shown in Figure 5a, the MLP model’s predictions on the training data are relatively high and are closer to the line of perfect prediction indicated in red. In addition, the results of the error analysis indicate an RMSE of 2.03 mV and an adjusted $R^{2}$ value of 0.956. These values imply that the MLP model is capable of nearly capturing the effects of changes in the training samples. In contrast, Figure 5b illustrates the MLP model’s performance on the test dataset. The MLP model exhibits a very minor decline in prediction capability, with an RMSE of 2.45 mV and an adjusted $R^{2}$ value of 0.952, when compared to the training dataset. Nevertheless, the resulting scatter plot reveals that the trained MLP is capable of predicting near the line of perfect prediction, highlighting the model’s capability to predict unseen data.

### 6.2. DDO to Access Superior PEMFC Performance

After evaluating the prediction capability of the MLP model to predict $V_{cell}$ for a given set of unseen data across a wide range of design variables within the confined design bounds, it was further linked to the PSO algorithm to address a DDO problem, which primarily focuses on predicting the maximum $V_{cell}$. 

**Figure 6:**
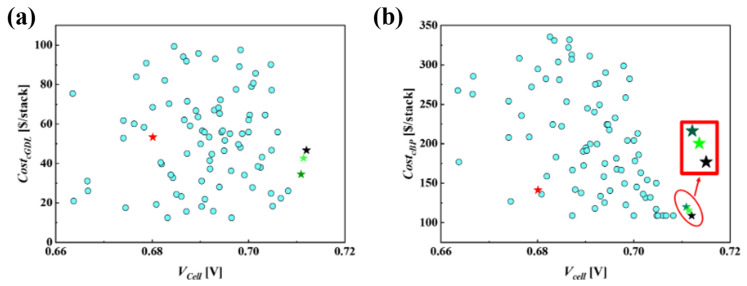
Evaluation of (**a**) $Cost_{cGDL}$ and $V_{cell}$ and (**b**) $Cost_{cBP}$ and $V_{cell}$ across various PEMFC designs at I=1.5 $A/cm^{2}$. This figure illustrates the sample data (●), baseline model (★) andoptimal design solutions obtained through DDO (★) and RBDO, including $RBDO_{1}\;$(★) and $RBDO_{2}\;$(★).

Figure 6 illustrates a scatter plot for the relationship between $V_{cell}$ and the cathode side material cost parameters, $Cost_{cGDL}$ and $Cost_{cBP}$, across various PEMFC designs, including the baseline, DDO, $RBDO_{1}$ and $RBDO_{2}$. As seen in Figure 6, the optimized PEMFC design via the MLP–PSO (DDO) method shows maximum $V_{cell}$ performance. Table 6 lists the design variables and corresponding $V_{cell}$ values for various PEMFC designs and cathode side material cost parameters, i.e., $Cost_{cGDL}$ and $Cost_{cBP}$. Particularly, as compared with the baseline design, DDO design shows a rise of 31 mV in $V_{cell}$. A drop in $V_{cell}$ performance is seen in the baseline design, which is due to $w_{ch}/w_{land}$ = 1:1, and a larger $d_{ch}$ corresponds to lower overall air velocity in the gas channel; these factors weaken oxygen transport and the removal of water that is accumulated inside the cathode GDL, while a thicker GDL limits oxygen transport along the through-plane direction (x). Interestingly, the increase in the $V_{cell}$ values of the DDO design also leads to a drop in $Cost_{cGDL}$ by 6.71 $/stack and $Cost_{cBP}$ by 32.64 $/stack. The resulting difference in cost parameters can be primarily attributed to the fact that the dimensions of the cathode side GDL and BP for the baseline design are significantly larger than the optimal values obtained through the DDO method. However, as noted in Table 6, the resulting DDO design predicted via MLP–PSO predicts design variables with $\delta_{gdl}=0.188\;mm$, $d_{ch}=0.3\;mm$, $w_{ch}=0.614\;mm$ and $w_{land}=0.31\;mm$ with $Cost_{cGDL}=46.68\$/stack$ and $Cost_{cBP}=108.81\;\$/stack$, where the $d_{ch}$ is predicted at the extreme end of the design space, in particular the lower bound which corresponds to the least possible cathode BP material cost. Take note that, for further discussions, the material cost parameters of the cathode side GDL and BP, as predicted by DDO, will be denoted as $Cost_{cGDL}^{DDO}$ and $Cost_{cBP}^{DDO}$, respectively. 

### 6.3. RBDO for Cathode, GDL and BP Material Costs

**Figure 7:**
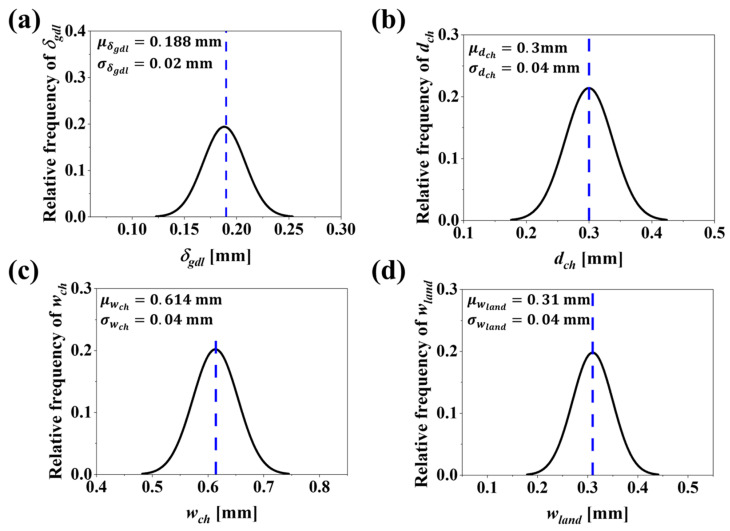
PDF plots with a 95% probability interval that corresponds to $Case_{1}$ for four design variables: (**a**) $\delta_{gdl}$, (**b**) $d_{ch}$, (**c**) $w_{ch}$ and (**d**) $w_{land}$. These plots account for uncertainties in design variables with standard deviations $\sigma_{\delta_{gdl}}=0.03\;mm$, $\sigma_{d_{ch}}=0.05\;mm$, $\sigma_{w_{ch}}=0.05\;$ mm and $\sigma_{w_{land}}=0.05\;mm.$.

In engineering design, DDO models have been widely used to maximize/minimize the cost function in consideration of constraints. Over the past decade, significant efforts have been dedicated to optimizing PEMFC designs and their components using DDO models. However, due to uncertainties in the production process, there is a need to transition to RBDO to ensure robust and reliable designs. As discussed in Section 6.2, DDO offers superior $V_{cell}$ values, with a reduction in $Cost_{cGDL}$ and $Cost_{cBP}$. When manufacturing uncertainties are incorporated into the design variables, the optimal solution often tends to deviate from the desired outcome, resulting in unreliable design. To address the limitations of DDO, an RBDO problem is developed, as detailed in Section 5, specifically Equations (43) and (44). In Table 7, the uncertainties that may arise during the manufacturing of the GDL and BPs have been considered for RBDO. These uncertainties are analyzed in two cases: $Case_{1}$ represents a high level of uncertainty, with standard deviations of $\sigma_{\delta_{gdl}}=0.03\;mm$, $\sigma_{d_{ch}}=0.05\;mm$, $\sigma_{w_{ch}}=0.05\;$ mm and $\sigma_{w_{land}}=0.05\;mm$. $Case_{2}$ represents a lower level of uncertainty, with standard deviations of $\sigma_{\delta_{gdl}}=0.01\;mm$, $\sigma_{d_{ch}}=0.03\;mm$, $\sigma_{w_{ch}}=0.03\;$ mm and $\sigma_{w_{land}}=0.03\;mm$. This distinction helps in understanding the impact of varying uncertainty levels on the reliability of the PEMFC design. Additionally, as seen in the table, the standard deviation of the GDL is typically lower than that of the BP. This is because the GDLs play a critical role in the transport of reactant gases and the removal of product water formed during fuel cell operation. Small variations in thickness and porosity can lead to deviations in the flow of reactants and products, thereby altering the overall performance of the fuel cell. In contrast, BPs primarily provide mechanical support and electrical connectivity in the fuel cell. While dimensional accuracy is necessary, slight changes in dimensional variations do not significantly alter $V_{cell}$, compared to the GDL. Additionally, the materials used in BPs provide high structural stability and are less sensitive to dimensional variations compared to porous GDLs. Therefore, the robustness of BPs offers a slightly relaxed edge in terms of manufacturing uncertainties compared to the GDL. Figure 7a–d show the probability distribution (PDF) with 95% probability intervals for the four design variables, $\delta_{gdl}$, $d_{ch}$, $w_{ch}\;$ and $w_{land}$ at the DDO optimal. These figures correspond to $Case_{1}$, where the design variables are subjected to uncertainties with standard deviations of $\sigma_{\delta_{gdl}}=0.03\;mm$, $\sigma_{d_{ch}}=0.05\;mm$, $\sigma_{w_{ch}}=0.05\;$ mm and $\sigma_{w_{land}}=0.05\;mm$, respectively. 

The objective of RBDO in the present study is to maximize the objective function $\;(V_{cell})$ considering uncertainties in manufacturing, as defined in $Case_{1}$ and $Case_{2}$, while ensuring that the constraints, i.e., $Cost_{cGDL}$ and $Cost_{cBP}$ do not violate the boundaries of significant performance metrices. Moreover, these cost parameters are accessed for reliability, ensuring that the PEMFC design remains robust under uncertainty. Therefore, setting the limits of these constraints is an important part of RBDO. As seen in Figure 3, the DDO design predicted via MLP–PSO is an optimal starting point for RBDO due to its significant advantages in both cost and performance compared to the baseline design. Specifically, $Cost_{cGDL}$ at the DDO is 46.68 $/stack, which is notably lower than 53.38 $/stack for the baseline design. Similarly, $Cost_{cBP}$ is at 108.81 $/stack, compared to 141.45 KRW/stack for the baseline design. These improvements demonstrate that the DDO not only reduces costs significantly but also enhances $V_{cell}$, making it a more optimal starting point for further RBDO. 

**Figure 8:**
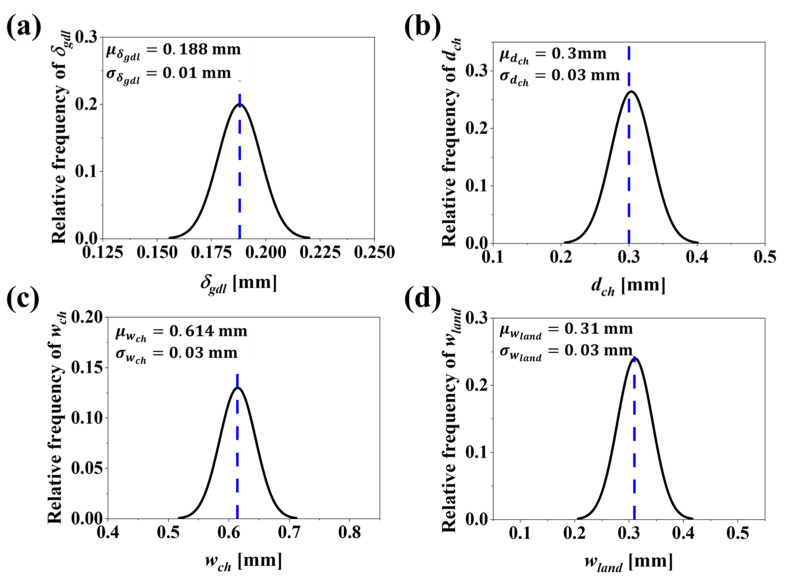
PDF plots with a 95% probability interval that corresponds to $Case_{2}$ for four design variables: (**a**) $\delta_{gdl}$, (**b**) $d_{ch}$, (**c**) $w_{ch}$ and (**d**) $w_{land}$. These plots account for uncertainties in design variables with standard deviations $\sigma_{\delta_{gdl}}=0.01\;mm$, $\sigma_{d_{ch}}=0.03\;mm$, $\sigma_{w_{ch}}=0.03\;$ mm and $\sigma_{w_{land}}=0.03\;mm.$

In Figure 7b and Figure 8b, at the DDO optimal, considering the uncertainties in design variables as defined in $Case_{1}$ and $Case_{2}$, variation in values for $d_{ch}$ is observed. Specifically, $d_{ch}$ falls significantly below the lower bound of the design space, set at $d_{ch}^{L}=0.3\;mm$. This deviation violates the design bounds. According to Equation (40), $Cost_{cBP}$ is directly proportional to $d_{ch}$. Therefore, $Cost_{cBP}$ will also fail to meet the design bounds for $d_{ch}$ values below $d_{ch}^{L}=0.3\;m$. This necessitates fixing the constraint value to $Cost_{cBP}\geq 108.81$ $/stack, i.e., $Cost_{cBP}\geq \;Cost_{cBP}^{DDO}$. The $Cost_{cGDL}$ is estimated based on Equation (38) and is directly proportional to $\delta_{gdl}$. As seen in Figure 7a,b, when uncertainties in $\delta_{gdl}$ as defined in $Case_{1}$ and $Case_{2}$ are considered, the variation in $\delta_{gdl}$, set at $\delta_{gdl}=0.188\;mm$, is well above the lower bound $\delta_{gdl}^{L}=0.03\;mm$. Therefore, the constraint for $Cost_{cGDL}$ is set as $Cost_{cGDL}\leq Cost_{cGDL}^{DDO}$.

**Figure 9:**
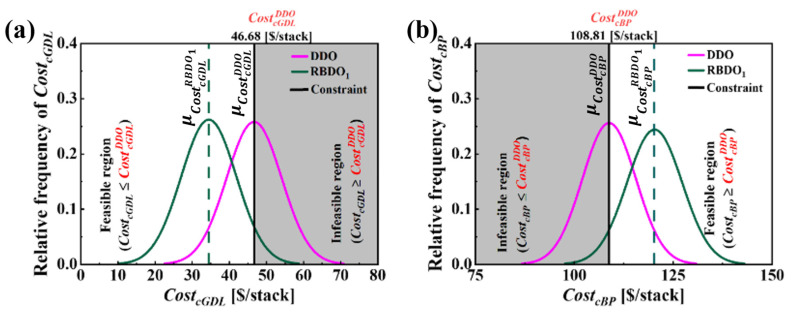
Comparison of PDF plots that correspond to $Case_{1}$ for (**a**) $Cost_{cGDL}$ and (**b**) $Cost_{cBP}$ for DDO ($\delta_{gdl}=0.188\;mm$, $d_{ch}=0.3\;mm$, $w_{ch}=0.614\;mm$ and $w_{land}=0.31\;mm$) and $RBDO_{1}$ ($\delta_{gdl}=0.139\;mm$, $d_{ch}=0.382\;mm$, $w_{ch}=0.855\;mm$ and $w_{land}=0.3\;mm$), incorporating uncertainties in design variables with standard deviations $\sigma_{\delta_{gdl}}=0.03\;mm$, $\sigma_{d_{ch}}=0.05\;mm$, $\sigma_{w_{ch}}=0.05$ mm and $\sigma_{w_{\mathrm{land}}}=0.05\;mm$. The operating current density at which $Cost_{cGDL}$ and $Cost_{cBP}$ is estimated is I=1.5 $A/cm^{2}$.

As shown in Figure 3, after defining the performance constraints, RBDO is initiated from the DDO considering uncertainties in design variables as defined in $Case_{1}$. The aim of the optimization process is to find a reliable optimal solution, referred as $RBDO_{1}$. Figure 9a,b compares the results of DDO and $RBDO_{1}$. As can be seen, the PDF plots at DDO indicate a clear violation of the constraints $Cost_{cGDL}\leq Cost_{cGDL}^{DDO}$ and $Cost_{cBP}\geq \;Cost_{cBP}^{DDO}$ with the distribution plots extending into the infeasible region depicted by gray shaded area. In contrast, $RBDO_{1}$ distribution plots are more spread out, reflecting a design strategy that accommodates the uncertainties while staying within the feasible cost region. Comparing and analyzing the detailed result listed in Table 8 reveals that the $Cost_{cGDL}$ in the DDO achieves a reliability of 49.87%, indicating that 50.13% of the designs are unreliable and fail to meet $Cost_{cGDL}\leq Cost_{cGDL}^{DDO}$. Conversely, the RBDO approach shows that the reliability for achieving $Cost_{cGDL}\leq Cost_{cGDL}^{DDO}$ at $RBDO_{1}$ is 95.0%. Regarding, $Cost_{cBP}$, at DDO the reliability is 50.0%, which indicates that 50.0% of the designs are unreliable and fall below $Cost_{cGDL}^{DDO}=46.68\;$$/stack. Furthermore, comparing the nominal values of $Cost_{cGDL}$ indicates that a reduction of 12.25 $/stack is achieved, attributed to the RBDO strategy of reducing the material cost of the cathode GDL. Consequently, the material cost for the cathode BP, $Cost_{cBP}$, inevitably increases by 11.18 $/stack , causing the reliability of meeting $Cost_{cBP}\geq \;Cost_{cBP}^{DDO}$ to increase from 50.0% to 94.99%. $RBDO_{1}$ has successfully navigated manufacturing uncertainties while consistently achieving a target reliability of 95% for both $Cost_{cGDL}$ and $Cost_{cBP}$ and these are well illustrated in Figure 8. 

Water management in PEMFCs is a critical process that involves balancing membrane hydration by avoiding flooding, particularly in the cathode GDL and CL [53]. Proper water management ensures that the PEM remains hydrated, which is essential for maintaining its ionic conductivity and overall cell performance. At the same time, it prevents excessive liquid water from accumulating in the GDL and porous electrodes, which can block reactant flow and hinder performance [54]. Effective water management not only optimizes the $V_{cell}$ but also enhances the durability of the fuel cell by preventing issues like membrane dehydration and flooding, which can cause irreversible damage [55]. As outlined in Table 6, comparing the results of design variables for the DDO and $RBDO_{1}$ reveals that the DDO design variables optimize the $V_{cell}$ by enhancing reactant distribution and water management, resulting in a superior $V_{cell}$ of 0.712 V. In contrast, the $RBDO_{1}$ design achieves a comparable $V_{cell}$ of 0.710 V by balancing efficiency and reliability, thereby aiming for a robust and reliable operation. This balance is crucial for maintaining high performance and extending the lifespan of PEMFCs, with the dimensions of the GDL and flow field playing a critical role in achieving proper water management.

**Figure 10:**
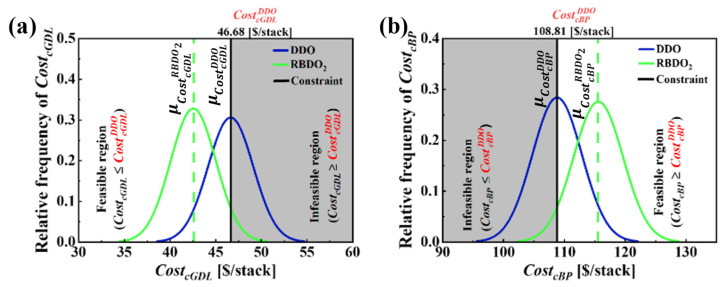
Comparison of PDF plots that corresponds to $Case_{2}$ for (**a**) $Cost_{cGDL}$ and (**b**) $Cost_{cBP}$ for DDO ($\delta_{gdl}=0.188\;mm$, $d_{ch}=0.3\;mm$, $w_{ch}=0.614\;mm$ and $w_{land}=0.31\;mm$) with standard deviation and $RBDO_{2}\;$($\delta_{gdl}=0.172\;mm$, $d_{ch}=0.349\;mm$, $w_{ch}=0.687\;mm$ and $w_{land}=0.3\;mm$), incorporating uncertainties in design variables with standard deviations $\sigma_{\delta_{gdl}}=0.01\;mm$, $\sigma_{d_{ch}}=0.03\;mm$, $\sigma_{w_{ch}}=0.03\;$ mm and $\sigma_{w_{land}}=0.03\;mm$. The operating current density at which $Cost_{cGDL}$ and $Cost_{cBP}$ is estimated is I=1.5 $A/cm^{2}$.

Assessing the effects of variability in uncertainty on PEMFC performance is of high interest. Analyzing multiple cases, such as $Case_{1}$ and $Case_{2},$ ensures that the PEMFC design is robust and reliable under different conditions. Figure 8 illustrates the PDF plots with a 95% probability interval that correspond to $Case_{2}$ for four design variables: (a) $\delta_{gdl}$, (b) $d_{ch}$, (c) $w_{ch}$ and (d) $w_{land}$. These plots account for uncertainties in design variables with standard deviations $\sigma_{\delta_{gdl}}=0.01\;mm$, $\sigma_{d_{ch}}=0.03\;mm$, $\sigma_{w_{ch}}=0.03\;$ mm and $\sigma_{w_{land}}=0.03\;mm.$ The RBDO approach aligns with previous descriptions, so our discussions are focused on how $RBDO_{2}$ affects PEMFC designs. Figure 10a,b display the distributions of $Cost_{cGDL}$ and $Cost_{cBP}$, where more than half of the distribution curve violates the constraints set at $Cost_{cGDL}\leq Cost_{cGDL}^{DDO}$ and $Cost_{cBP}\geq \;Cost_{cBP}^{DDO}$. Comparing the values of DDO and $RBDO_{2}$ shows that the RBDO method predicts a $Cost_{cGDL}$ much lower than that of DDO, and the reduction in cost is 4.09 $/stack with a reliability of 95.02%. Regarding $Cost_{cBP},$ the RBDO method shows an inevitable cost increase of 6.71 $/stack while achieving a reliability target of 95%. Comparing and analyzing the effects of varying uncertainty as defined in $Case_{1}$ and $Case_{2}$ reveals that the RBDO method tries to achieve the target reliability of 95% in both cases. In addition, $RBDO_{1}$ and $RBDO_{2}$ show how different levels of uncertainty affect the design variables and performance. $RBDO_{1}$, with higher variability, results in a more conservative design with lower costs but slightly lower $V_{cell}$ values. In contrast, $RBDO_{2}$, with lower variability, achieves a more optimized design with slightly higher costs but similar $V_{cell}$ values. Both approaches maintain comparable cell voltages, demonstrating robust performance despite the differences in design strategies.

## 7. Conclusions

This study presents a methodology for optimizing the four key PEMFC design variables, i.e., $\delta_{gdl}$, $d_{ch}$, $w_{ch}\;$ and $w_{land}$. We employed two optimization strategies, namely DDO and RBDO, to evaluate and compare effectiveness. At first, an MLP model was developed based on the results from a comprehensive multi-scale, two-phase, 3D numerical PEMFC model. The predictive accuracy of the MLP was evaluated on the test set of 15 design samples using the RMSE and adjusted $R^{2}$, with values of RMSE = 2.45 mV and adjusted $R^{2}\;$= 0.952. The MLP was integrated with a PSO to perform DDO, which identified a design that improved $V_{cell}$ by 31 mV at a current density of 1.5 A/cm^2^. This superior $V_{cell}$ is primarily due to its optimized design parameters by reducing the design variables as compared to the baseline case. These optimized dimensions enhance the flow distribution, leading to a higher $V_{cell}$ of 0.712 V. Given the manufacturing variability in the cathode GDL and BP, these uncertainties were modeled using statistical distributions, and RBDO was conducted. The RBDO results indicated that designs deemed optimal in DDO contexts failed to meet the cost constraint, $Cost_{cGDL}\leq Cost_{cGDL}^{DDO}$ and $Cost_{cBP}\geq \;Cost_{cBP}^{DDO}$, illustrating the need for RBDO to enhance robustness in the manufacturing process by optimizing design variables to achieve over 95% reliability in ${Cost}_{cGDL}$ and ${Cost}_{CBP}$. The RBDO approach effectively balances efficiency and reliability, achieving a target reliability of 95% for both ${Cost}_{cGDL}$ and ${Cost}_{CBP}$. $RBDO_{1}$, with higher variability, results in a more conservative design with lower costs, while $RBDO_{2}$, with lower variability, achieves a more optimized design with slightly higher costs. Both designs maintain comparable $V_{cell}$ values, demonstrating robust performance despite different design strategies. 

## Figures and Tables

**Table 6:** Comparison of design variables and corresponding cell voltage $\left(V_{cell}\right)$ and nominal material costs of the cathode GDL and BP across various PEMFC designs: baseline, DDO, $RBDO_{1}$ and $RBDO_{2}$.

Parameters	Baseline Design	Optimization Approach		
		DDO	$RBDO_{1}$	$RBDO_{2}$
$\delta_{gdl}$, [mm]	0.215	0.188	0.139	0.172
$d_{ch}$, [mm]	0.54	0.300	0.382	0.349
$w_{ch}$, [mm]	1	0.614	0.855	0.687
$w_{land}$, [mm]	1	0.310	0.300	0.300
$V_{cell},\;[V]$	0.681	0.712	0.710	0.711
$Cost_{cGDL}$ [$/stack]	53.38	46.68	34.43	42.59
$Cost_{cBP}$ [$/stack]	141.45	108.81	119.99	115.52

**Table 7:** Distribution of design variables with mean  (μ) and standard deviations  (σ) for different cases.

Design Variables	Distribution	Mean (***μ***) [mm]	Standard Deviation (σ), [mm]	
		DDO	$Case_{1}$	$Case_{2}$
GDL thickness ($\delta_{gdl}$), [mm]	Normal	0.188	0.03	0.01
Channel depth ($d_{ch}$), [mm]	Normal	0.3	0.05	0.03
Channel width ($w_{ch}$), [mm]	Normal	0.614	0.05	0.03
Land width ($w_{land}$), [mm]	Normal	0.310	0.05	0.03

**Table 8:** A summary of various performance metrices for the cost parameters $Cost_{cGDL}$ and $Cost_{cBP}$, including the nominal and mean  (μ) value, standard deviation (σ) and the reliability assessments for the two cases, $Case_{1}$ and $Case_{2}$, as predicted through DDO and RBDO approaches.

Case	Parameter	Optimization Approach							
		DDO				RBDO			
		Reliability [%]	Nominal Value [$/Stack]	Mean Value (μ) [$/Stack]	Standard Deviation (σ) [$/Stack]	Reliability [%]	Nominal Value [$/Stack]	Mean Value (μ) [$/Stack]	Standard Deviation (σ) [$/Stack]
1	$Cost_{cGDL}$ [$/stack]	49.87	46.68	46.68	7.45	95.00	34.43	34.42	7.44
	$Cost_{cBP}$ [$/stack]	50.00	108.81	108.81	6.78	94.99	119.99	120.0	6.80
2	$Cost_{cGDL}$ [$/stack]	49.87	46.68	46.68	2.48	95.02	42.59	42.58	2.48
	$Cost_{cBP}$ [$/stack]	50.00	108.81	108.81	4.07	94.99	115.52	115.51	4.08

## Data Availability

The original contributions presented in the study are included in the article, further inquiries can be directed to the corresponding author.

## Nomenclature

*a*	Ratio of active surface area per unit electrode volume: m^2^/m^3^ or water activity
*A*	Area, m^2^
*C*	Molar concentration of species, mol/m^3^
***d***	Vector of design variables or solution of a deterministic optimization problem; $d={\left[d_{1},d_{2},\ldots ,d_{n}\right]}^{T}$
*D*	Species diffusivity, m^2^/s
*E*	Activation energy, kJ/mol
*EW*	Equivalent weight of a dry membrane, kg/mol
*f*	Objective function that needs to be minimized or maximized in the optimization problem
*F*	Faraday’s constant, 96,487 C/mol
*G*(**X**)	Constraint condition for the j-th constraint; the design is considered “fail” if *G*(**X**) fails to meet the constraint condition
*i*_0_	Exchange current density, A/cm^2^
*i*_*d*_	Density estimation parameter
*I*	Operating current density, A/cm^2^
*j*	Transfer current density, A/cm^3^,
J	Total number of constraint functions in the optimization problem
*k*	Thermal conductivity, W/m·K, or Relative permeability, or index representing the specific objective function in the optimization problem
*K*	Hydraulic permeability, m^2^
*L*	Amount of loading, ${mg/cm}^{2}$
*n*	Number of electrons transferred in the electrode reaction
*N*	Number of design variables
*MW*	Molecular weight, kg/mol
*MSE*	Mean squared error
P	Pressure, Pa,
*P*(●)	Probability function
*RMSE*	Root mean squared error
*s*	Liquid saturation
*S*	Source term in the transport equation
*t*	Time
*T*	Temperature, K
$\vec{u}$	Fluid velocity and superficial velocity in a porous medium, m/s
*V*	Voltage, V or Volume, $m^{3}$
**X**	Vector of the design variables in the optimization problem
$X_{i}$	Lower or upper bound of the i-th design variable
x	Input variable
v	Observed response
$\hat{v}$	Predicted response
$\bar{v}$	Mean value of the observed data
z	Transport resistance coefficient

## Greek Symbols

*α*	Transfer coefficient
β	Weight coefficient
*γ*	Reaction order
*δ*	Thickness, m
*ε*	Volume fraction or error
*η*	Surface overpotential, V
θ	Contact angle of the gas diffusion layer
λ	Water content
μ	Mean value of random design variables **X**, $\mu ={\left[\;\mu_{1},\;\mu_{2},\;\ldots ,\;\mu_{n}\right]}^{T}$
*κ*	Proton conductivity, S/m
ϕ	Phase potential, V
*ρ*	Density, kg/m
*σ*	Electronic conductivity, S/m
*τ*	Viscous shear stress, N/m^2^
*ξ*	Stoichiometry flow ratio
*Ω*	Oxygen transport resistance

## Superscripts

*a*	Anode
*aCL*	Anode catalyst layer
*c*	Cathode
*C*	Carbon
*CL*	Catalyst layer
*cCL*	Cathode catalyst layer
*ch*	Gas channel
*e*	Electrolyte
*ECSA*	Electro chemical active surface area
*gdl*	Gas diffusion layer
Fj	Index representing the failure of the j-th constraint
*I/C*	Ionomer to carbon weight ratio
*i*	Species or index representing the lower or upper bound of the *N*-th design variable
*in*	Channel inlet
int	Interface
*j*	Index representing the specific constraint function in a problem with multiple constraints
*k*	Index representing the specific objective function in the optimization problem
*MEA*	Membrane electrode assembly
*mem*	Membrane
*min*	Minimum
*N*	Number of design variables
*nd*	*n*-th random design variable
*Pt/C*	Weight ratio of platinum to carbon
*Pt*	Platinum
*s*	Solid, surface
*T*	Temperature
*u*	Momentum equation
*w*	Water
0	Initial conditions or standard conditions, i.e., 298.15 K and 101.3 kPa (1 atm)

## Abbreviations

BP	Bipolar plate
CFD	Computational fluid dynamics
CL	Catalyst layer
DDO	Deterministic design optimization
DKG	Dynamic Kriging
DoE	Design of experiments
EMS	Energy management strategy
FC	Fuel cell
FCHEV	Fuel cell hybrid electric vehicle
GDL	Gas diffusion layer
HOR	Hydrogen oxidation reaction
MLP	Multi-layer perceptron
ORR	Oxygen reduction reaction
PSO	Particle swarm optimization
$M^{2}$	Multiphase mixture
MCS	Monte Carlo simulation
MEA	Membrane electrode assembly
MSE	Mean square error
RAMDO	Reliability Analysis & Multidisciplinary Design Optimization
RBDO	Reliability-based design optimization
RMSE	Root mean square error
USA	United States of America
